# Mechanism of Cinnamaldehyde in Alleviating *Staphylococcus aureus*-Induced Mammary Inflammatory Response

**DOI:** 10.3390/ani16172742

**Published:** 2026-09-02

**Authors:** Xiaohui Chen, Jingge Wang, Huiyuan Ma, Wenbin Jiang, Guiqin Wang

**Affiliations:** 1 College of Resources Environment & Life Sciences, Ningxia Normal University, Guyuan 756099, China; cxh07232021@163.com (X.C.);; 2 College of Animal Science and Technology, Ningxia University, Yinchuan 750021, China; 3 Key Laboratory of Soil Ecological Health and Microbial Regulation, Ningxia Normal University, Guyuan 756099, China

**Keywords:** cinnamaldehyde, *Staphylococcus aureus*, mouse mastitis, cell inflammation

## Abstract

CA, a major bioactive component of cinnamon essential oil, possesses broad-spectrum antibacterial, anti-inflammatory, and antioxidant activities and is widely recognized as a safe food additive. In the context of exploring natural plant extracts for the prevention and alleviation of bovine mastitis, it is noteworthy that research and clinical reports on the use of cinnamon essential oil for bovine mastitis are relatively limited. In this study, *S. aureus* isolates recovered from subclinical bovine mastitis were used to establish a mastitis infection model, and the antioxidant and anti-inflammatory effects of CA were preliminarily investigated in both mouse mastitis models and mammary epithelial cells (MAC-T). Studies have demonstrated that in a mouse model of mastitis, CA reduces inflammatory cell infiltration, decreases myeloperoxidase (MPO) activity, and inhibits *S. aureus* colonization in mammary tissue. CA alleviates oxidative stress by lowering malondialdehyde (MDA) levels, enhancing superoxide dismutase (SOD) and glutathione peroxidase (GSH-Px) activities, and activating the Nrf2/HO-1/xCT antioxidant pathway. In addition, CA suppresses the activation of the TLR4/NF-κB signalling pathway and modulates the expression of Bax and Bcl-2, accompanied by reduced levels of pro-inflammatory cytokines, including tumour necrosis factor-alpha (TNF-α), interleukin-1 beta (IL-1β), and interleukin-6 (IL-6), ultimately ameliorating mastitis in mice. In the MAC-T cell infection model, CA significantly inhibited *S. aureus* adhesion and invasion and decreased the production of pro-inflammatory cytokines and mediators. CA also activated the Nrf2/HO-1/xCT antioxidant pathway, which led to decreased intracellular reactive oxygen species (ROS), concurrently inhibited the TLR4/NF-κB pathway, and downregulated apoptotic protein expression, ultimately attenuating inflammation in MAC-T cells. Collectively, these findings demonstrate that CA exerts protective effects against inflammation and oxidative damage in both mammary tissues and epithelial cells infected with *S. aureus*. Thus, this study provides a theoretical and practical basis for further exploring CA as a candidate therapy for bovine mastitis.

## Simple Summary

Cinnamaldehyde (CA) effectively inhibits *Staphylococcus aureus* (*S. aureus*) colonisation in mammary tissue, reduces inflammation and apoptosis by blocking the toll-like receptor 4 (TLR4)/nuclear factor kappa B (NF-κB) pathway, and alleviates oxidative stress through activation of the nuclear factor erythroid 2-related factor 2 (Nrf2)/heme oxygenase-1 (HO-1)/cystine/glutamate antiporter subunit (SLC7A11/xCT) antioxidant system. *In vitro*, CA prevents bacterial adhesion and invasion and lowers intracellular ROS levels. These findings indicate that CA possesses potential for both preventing and alleviating bovine mastitis.

## 1. Introduction

Mastitis is among the most economically devastating diseases affecting the global dairy industry, representing a major threat to sustainable development and resulting in considerable economic losses [1]. Bovine mastitis is an infectious disease predominantly caused by pathogenic bacteria, including *Staphylococcus aureus* (*S. aureus*), *Escherichia coli* (*E. coli*), and *Streptococcus agalactiae* (*S. agalactiae*). The condition often results in decreased milk yield and quality, elevated somatic cell counts (SCCs), and pathological alterations in mammary gland tissue. Research indicates that these pathogens typically invade mammary tissue through the teat canal, where they undergo rapid proliferation and establish infection [2]. This invasion triggers an increase in circulating inflammatory mediators, which promotes the recruitment of mast cells and macrophages and subsequently enhances the production of pro-inflammatory cytokines. These cytokines further attract neutrophils from the bloodstream into the mammary tissue, thereby exacerbating the inflammatory response. Prolonged or severe inflammation can result in substantial damage to the mammary gland, including tissue necrosis and atrophy [3,4]. Among these pathogens, *S. aureus* is a major causative agent of subclinical mastitis in dairy cows. It possesses the ability to form biofilms, thereby facilitating its adhesion to and invasion of bovine mammary epithelial cells. Furthermore, *S. aureus* can induce the formation of autophagosomes, allowing it to evade immune clearance and thus promoting persistent infections and chronic disease progression [5]. In addition to bovine mastitis, *S. aureus* is also an important zoonotic pathogen. It can cause a variety of infections, including skin infections, pneumonia, endocarditis, septicaemia, and toxigenic diseases. Therefore, it has significant public health importance [6]. 

It is well known that prophylactic antibiotic therapy is one of the most effective approaches to reducing intramammary infections in dairy cows. However, the efficacy of antibiotic treatment is limited, and over-reliance on antibiotics promotes the emergence of antimicrobial resistance (AMR) [7,8,9,10]. *S. aureus* is known to develop resistance to multiple antibiotics, methicillin-resistant *S. aureus* (MRSA) infections are zoonotic, and thus only a limited number of antibiotics can be used for long-term treatment [11]. Moreover, in subclinical bovine mastitis, β-lactamase production is more commonly observed among *S. aureus* isolates than in clinical isolates, and these strains exhibit resistance to multiple classes of antibiotics [12]. Prolonged use of antibiotics not only leads to drug residues in meat and dairy products but also induces the emergence of “superbugs” [13]. As a result, the development of natural and non-toxic alternatives to antibiotics has attracted growing research interest and public concern. Numerous studies have demonstrated that certain plant-derived compounds possess substantial potential as antibiotic alternatives, and some of their by-products can also be used as feed additives [14,15]. Natural products have garnered increasing attention because of their antibacterial and anti-inflammatory activities [16,17]. The search for plant extracts containing these bioactive compounds has emerged as a promising strategy for preventing and alleviating bovine mastitis. 

Essential oils are hydrophobic, aromatic, volatile oily liquids, comprising primarily aldehydes, phenols, alcohols, acids, ketones, terpenes, and aromatic compounds [18]. Among these, aldehydes and phenols are the most bioactive constituents, with CA—an aromatic aldehyde—being the principal active component of cinnamon essential oil [19]. CA is a naturally occurring antimicrobial agent that exhibits potent antibacterial, anti-inflammatory, antioxidant, and antitumor properties [20]. Furthermore, CA demonstrates outstanding antibacterial activity and safety in the food industry, where it is widely used as a preservative to inhibit foodborne pathogens and spoilage microorganisms [21]. In addition, it holds considerable therapeutic potential for alleviating chronic inflammation and oxidative stress-related diseases [22].

Previous findings from this study have demonstrated that CA effectively inhibits the growth and proliferation of *S. aureus*, exhibiting potent antibacterial activity [23]. However, it remains unclear whether CA can attenuate *S. aureus*-induced mammary gland inflammation and what signalling pathways are involved in this process. To further elucidate the anti-inflammatory and antioxidant mechanisms of CA, the present study established both an *in vivo* mouse infection model and an *in vitro* infection model using *S. aureus*-induced MAC-T cells. RT-qPCR and Western blot analyses were employed to investigate the molecular mechanisms by which CA mitigates *S. aureus*-induced mastitis.

## 2. Materials and Methods

### 2.1. Reagents

The cinnamaldehyde, with a purity exceeding 95%, was purchased from Yuan Ye (Shanghai, China). The MAC-T bovine mammary epithelial cell line was obtained from Haixing Biotechnology Co., Ltd. (Suzhou, China). The DCFH-DA ROS fluorescent probes were purchased from Solarbio (Beijing, China). The Cell Counting Kit-8 (CCK-8) was provided by Subcoin (Wuhan, China). The Annexin V-FITC Apoptosis Detection Kit was obtained from Beyotime (Shanghai, China). The SPARKscript II All-in-One RT SuperMix for qPCR Kit was obtained from Cisco Jet Biotechnology Co., Ltd. (Jinan, China). Enzyme-linked immunosorbent assay (ELISA) kits of IL-6, IL-10, and TNF-α were purchased from Jiubang Biotechnology Co., Ltd. (Quanzhou, China). Enzyme-linked immunosorbent assay (ELISA) kits of SOD, MDA and GSH-Px were purchased from Pumei Biology Co., Ltd. (Wuhan, China). ELISA kits of MPO were purchased from Solarbio (Beijing, China).

### 2.2. Animal Handling and Experimental Groups

Thirty-five Kunming mice (7–8 weeks old, 20–25 g) were procured from the Lanzhou Veterinary Research Institute, Chinese Academy of Agricultural Sciences, and used within three days of arrival. The animals were randomly allocated into five groups (*n* = 7 per group): a control group, an *S. aureus* infection group (SA group), and three treatment groups receiving low (5 mg/kg), medium (25 mg/kg), or high (50 mg/kg) doses of CA. All animal experiments involving mice were approved by the Animal Ethics Committee of Ningxia University.

Prior to experimentation, all mice underwent a one-week acclimation period under controlled environmental conditions (24 ± 1 °C, 65% relative humidity) with free access to standard chow and sterile water. CA was diluted with sterile phosphate-buffered saline (PBS) to achieve final concentrations of 5, 25, and 50 mg/kg. Anaesthesia was induced by intraperitoneal injection of pentobarbital sodium (40 mg/kg). Following anaesthesia, approximately 1 mm of the nipple tip was removed to expose the mammary duct, after which 100 μL of a fresh overnight *S. aureus* suspension (1 × 10^6^ CFU/mL) was administered slowly via microsyringe. The control group was administered an equivalent volume of sterile PBS. 12 h post-infection, CA was administered intraperitoneally three times at 6 h intervals. After 24 h of model establishment, mice were intraperitoneally injected with pentobarbital sodium (150–200 mg/kg). When the mice showed slowed breathing and disappearance of the righting reflex, cervical dislocation was immediately performed. After confirming that the heartbeat had stopped, serum and mammary gland tissues were collected and stored at −80 °C for subsequent analysis. All experimental procedures were conducted in full compliance with institutional animal welfare guidelines and ethical regulations. 

### 2.3. Histopathological Assessment

Mammary gland tissues from mice were fixed in 4% paraformaldehyde and embedded in paraffin. Tissue sections approximately 4–5 μm in thickness underwent standard histological processing, comprising dehydration, clearing, embedding, sectioning, mounting, and haematoxylin and eosin (H&E) staining. The sections were subsequently dehydrated, cleared, and coverslipped, after which histopathological alterations were examined under a light microscope. Histological evaluation was performed in a randomized manner using a scoring system, with scores defined as: 0, no injury; 1, mild injury; 2, moderate injury; 3, severe injury; and 4, extremely severe injury.

### 2.4. MPO Activity Assay

Approximately 0.1 g of mammary gland tissue was weighed, finely minced, and homogenized in an appropriate volume of sterile PBS at low temperature. After centrifugation of the homogenate, the supernatant was harvested. MPO activity was measured with a commercial assay kit in accordance with the manufacturer’s instructions. The inhibitory effect of CA on MPO activity in *S. aureus*-induced mastitis was evaluated by statistical analysis.

### 2.5. S. aureus Colonization in Mammary Tissue

Additionally, to assess the effect of CA on *S. aureus* colonization in mammary tissue, approximately 0.1 g of mammary tissue was homogenized in sterile PBS at a low temperature. The resulting homogenate was serially diluted in PBS at 10^2^–10^3^-fold gradients, and 100 μL of each dilution was spread onto Mueller–Hinton agar (MHA) plates, followed by overnight incubation at 37 °C for 12 h.

### 2.6. Assessment of the Expression of Inflammatory Mediators

Serum samples from each experimental group were collected, and the concentrations of the pro-inflammatory cytokines IL-6, IL-1β, and TNF-α were quantified using ELISA kits in accordance with the manufacturer’s instructions. Simultaneously, RT-qPCR was performed to determine the mRNA expression levels of inflammatory cytokines in mouse mammary tissue. The primer sequences used in this study are presented in Table 1.

### 2.7. Assessment of Oxidative Stress Marker Expression

Approximately 0.1 g of mouse mammary tissue was weighed, minced, and homogenized in an appropriate volume of sterile PBS in a centrifuge tube at low temperature. The tissue homogenate was subjected to centrifugation, and the supernatant was collected. The expression levels of the oxidative stress biomarkers SOD, MDA, and GSH-Px were determined with commercial ELISA kits.

### 2.8. Cell Culture, Preparation, and Treatment

MAC-T cells were seeded into 25 cm^2^ culture flasks containing Dulbecco’s modified Eagle medium (DMEM) supplemented with 10% foetal bovine serum (FBS) and 100 IU/mL penicillin–streptomycin and incubated at 37 °C in a 5% CO_2_ atmosphere. When cells reached approximately 80% confluence, they were passaged. Meanwhile, *S. aureus* was grown to the logarithmic phase, collected, washed three times with sterile PBS, and resuspended in sterile PBS. CA was dissolved in dimethyl sulfoxide (DMSO) to a final concentration of 100 mM. 

Upon reaching 70% confluence, MAC-T cells received different treatments. The control group was maintained in serum-free DMEM containing penicillin–streptomycin. The *S. aureus* infection group (SA group) was infected with the infection medium (DMEM containing 5% FBS and penicillin–streptomycin) at a multiplicity of infection (MOI) of 100 for 6 h, as described previously. The drug pretreatment group (SA + CA group) was pretreated with CA-containing medium (DMEM with 10% FBS and penicillin–streptomycin) for 24 h. After removal of the medium, the cells were infected with infection medium and incubated at 37 °C in a 5% CO_2_ atmosphere for 6 h.

### 2.9. Assessment of Cell Viability

The effect of CA on the viability of MAC-T cells was assessed using a Cell Counting Kit-8 (CCK-8) assay kit. MAC-T cells at 80% confluence were seeded in a 96-well plate at 100 μL per well. The medium was then replaced with DMEM containing varying concentrations of CA, and the cells were incubated for 24 h. Following incubation, 10 μL of CCK-8 solution was added to each well, and the plates were incubated for a further 1 h. Cell viability was determined by measuring the optical density at 450 nm using a multimode microplate reader (SuPerMax 3100, Shanghai Flash Spectrum Biotechnology Co., Ltd., Shanghai, China). 

### 2.10. Assessment of S. aureus Adhesion and Invasion

To assess *S. aureus* adhesion, MAC-T cells were seeded into six-well plates at a density of approximately 1 × 10^5^ cells/mL and cultured at 37 °C in a 5% CO_2_ atmosphere until reaching 70% confluence. The medium was removed, and the cells were exposed to treatment media according to the experimental design. Following trypsin digestion, the cell pellet was collected, serially diluted at 10^2^–10^3^-fold dilutions in PBS, and 100 μL of each dilution was plated onto MHA agar and colonies were counted. 

To assess *S. aureus* invasion, MAC-T cells were infected with *S. aureus* according to the experimental design. Following infection, the cells were washed three times with PBS and incubated with 100 μg/mL gentamicin for 45 min to eliminate extracellular bacteria. The cells were then washed three times with PBS, and 0.1% (*v*/*v*) Triton X-100 was added to lyse the monolayer at 4 °C for 20 min. The resulting cell suspension was collected, serially diluted at 10^2^–10^3^-fold in PBS, and 100 μL of each dilution was plated onto MHA agar and the colonies were counted.

### 2.11. Assessment of Intracellular ROS Levels

MAC-T cells were seeded in 24-well plates and cultured for the designated time. Following treatment, the culture medium was removed, and the cells were incubated with DCFH-DA (10 μmol/L) diluted in serum-free medium at 37 °C in the dark for 20 min. After three washes with PBS to remove excess probes, fluorescence images were captured using a fluorescence microscope (Leica, Teaneck, NJ, USA). The average fluorescence intensity of DCFH-DA in each image was quantified using Image J (1.51j) software after background subtraction.

### 2.12. Assessment of the Expression of Cell Adhesion Molecules and Inflammatory Mediators

MAC-T cell samples were collected and RNA was extracted by the Trizol method. Reverse transcription and real-time quantitative PCR (RT-qPCR) were performed using the SPARKscript II All-in-one RT Super Mix for qPCR Kit. Primer sequences used in this study are presented in Table 2.

### 2.13. Western Blot

For protein extraction, mouse mammary tissue samples (approximately 0.05 g) were weighed and placed into 1.5 mL centrifuge tubes. Pre-cooled RIPA lysis buffer and protease inhibitor were added, followed by incubation on ice for 30 min to facilitate lysis. The supernatant was collected by centrifugation, discarding the pellet. In parallel, MAC-T cells from each treatment group were lysed with 250 μL of pre-cooled RIPA lysis buffer containing protease inhibitors, incubated on ice for 30 min, and centrifuged to collect the supernatants. Total protein from both tissue and cell samples was extracted using a complete protein extraction kit (Nanjing Kaiji Biotechnology Development Co., Ltd., Nanjing, China) and quantified with a protein quantification kit (BCA; Thermo Fisher, Waltham, MA, USA). Medium amounts of proteins from each sample were separated via sodium dodecyl sulphate polyacrylamide gel electrophoresis and transferred to polyvinylidene fluoride membranes. Membranes were blocked at room temperature with 5% skim milk containing tween-20 or 3% bovine serum albumin (BSA) (Solarbio Science and Technology Co., Ltd., Beijing, China) for 2 h at room temperature and then incubated with specific primary antibodies at 4 °C for 12 h. Membranes were incubated with the relevant horseradish peroxidase (HRP)-labelled secondary antibodies at room temperature for 2 h and protein bands were visualized using an enhanced chemiluminescence (ECL) detection solution (Beyotime, P0018AS, Shanghai, China). The primary antibodies obtained from the Proteintech group (Wuhan, China) were anti-TLR4 (66350-1-Ig, 1:1000), anti-p65 (10745-1-AP, 1:1000), anti-Nrf2 (16396-1-AP, 1:2000), anti-HO-1 (81281-1-RR,1:5000), anti-xCT (26864-1-AP, 1:1000), anti-Bax (50599-2-Ig, 1:2000), anti-Bcl2 (26593-1-AP, 1:500), anti-Caspase3 (19677-1-AP, 1:1000), and anti-Caspase9 (10380-1-AP, 1:500). The primary antibodies obtained from Abclonal (Wuhan, China) were anti-GAPDH (A19056, 1: 1000), and anti-β-actin (AC006, 1: 10,000). The primary antibody obtained from Ablmart (Shanghai, China) was anti-p-p65 (TP70621, 1:1000). The primary antibody obtained from Seville (Wuhan, China) was anti-Cytc (GB11080-100, 1:1000). The secondary antibodies were HRP-goat anti-rabbit IgG (AS014, 1:5000) and HRP-goat anti-mouse IgG (AS003, 1:5000).

### 2.14. Statistical Analysis

Data were validated in three independent experiments, and the gray values of Western blot bands of proteins were obtained using Image J (1.51j) software and analysed by GAPDH or β-actin-relative quantitative normalization. Experimental data were analysed by one-way analysis of variance using OriginPro 2018 (64-bit) software. Data are expressed as mean ± standard deviation (X ± SD). The significance of differences among groups is denoted by lowercase letters, where groups sharing the same letter are not significantly different, whereas groups with different letters indicate a statistically significant difference at *p* < 0.05.

## 3. Results

### 3.1. Effect of CA on Histopathological Alterations in Mouse Mastitis

**Figure 1:**
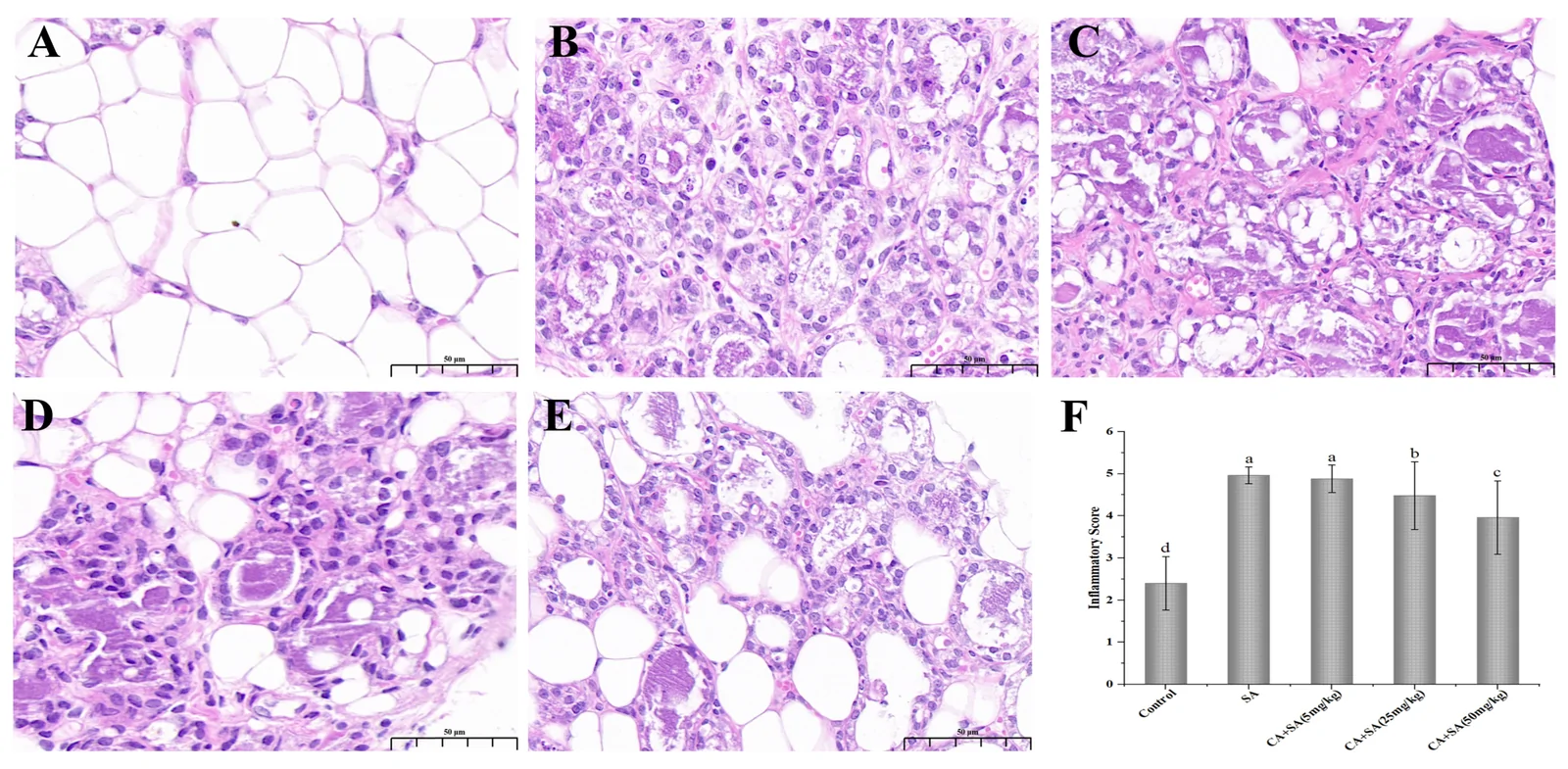
Effect of CA on histopathological changes of mammary gland in mouse. Note: (**A**) Control group, (**B**) *S. aureus* infection group, (**C**) CA low-dose group (5 mg/kg), (**D**) CA medium-dose group (25 mg/kg), (**E**) CA high-dose group (50 mg/kg), *n* = 7 mice per group, (**F**) Histopathological scoring of mouse mammary gland. The significance of differences among groups is denoted by lowercase letters, where groups sharing the same letter are not significantly different, whereas groups with different letters indicate a statistically significant difference at *p* < 0.05.

The impact of CA on pathological changes in mouse mammary tissue was examined under an optical microscope. In the control group, mammary tissue was intact without apparent histopathological changes. The mammary acini were intact, duct boundaries were sharply demarcated, and no inflammatory cells were observed within the acini (Figure 1A). In contrast, mammary tissue from the *S. aureus* infection group exhibited significant damage, characterized by extensive infiltration of inflammatory cells, increased neutrophil infiltration, thickened duct walls, and poorly defined duct boundaries (Figure 1B). In the CA-treated groups, infiltration of inflammatory cells was markedly reduced, particularly in the high-dose CA group, and the boundaries of the mammary acini appeared more distinct. These results indicate that CA effectively mitigated histopathological damage in mouse mammary tissue induced by *S. aureus* infection (Figure 1C–E). Furthermore, to verify the extent of tissue damage, histopathological changes in mouse mammary glands were assessed by scoring the number of infiltrating inflammatory cells. The pathological grades were scored (Figure 1F) and the results were consistent with the histopathological observations.

### 3.2. Effect of CA on MPO Activity in Mouse Mastitis

**Figure 2:**
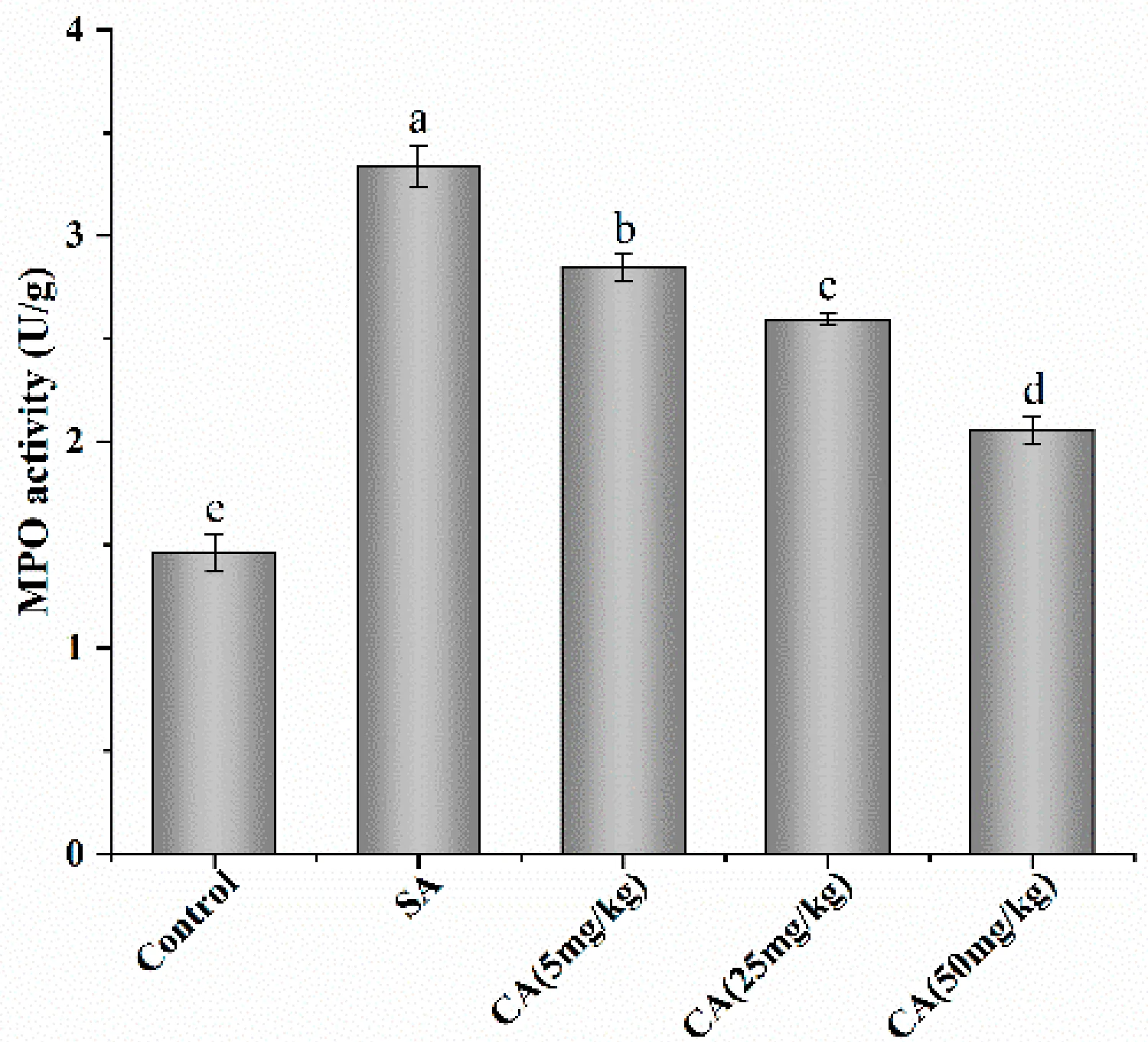
The effect of CA on MPO activity in mouse mastitis. Note: different letters indicate significant differences (*p* < 0.05), *n* = 7 mice per group. The significance of differences among groups is denoted by lowercase letters, where groups sharing the same letter are not significantly different, whereas groups with different letters indicate a statistically significant difference at *p* < 0.05.

MPO is a key enzyme in neutrophils, and its activity reflects neutrophil infiltration. Assessment of MPO activity serves as a valuable indicator for evaluating neutrophil infiltration in mammary tissue and assessing the severity of inflammation. In this study, the effect of CA on MPO activity in a mouse mastitis was evaluated. The results demonstrated that, compared with the control group, MPO activity in the mammary tissue of the *S. aureus*-infected mice was significantly elevated. In contrast, MPO activity in the CA-treated groups was decreased in a dose-dependent manner relative to the infection group (*n* = 7 mice per group, *p* < 0.05, Figure 2). 

### 3.3. Effect of CA on the Colonization of S. aureus in Mouse Mastitis

**Figure 3:**
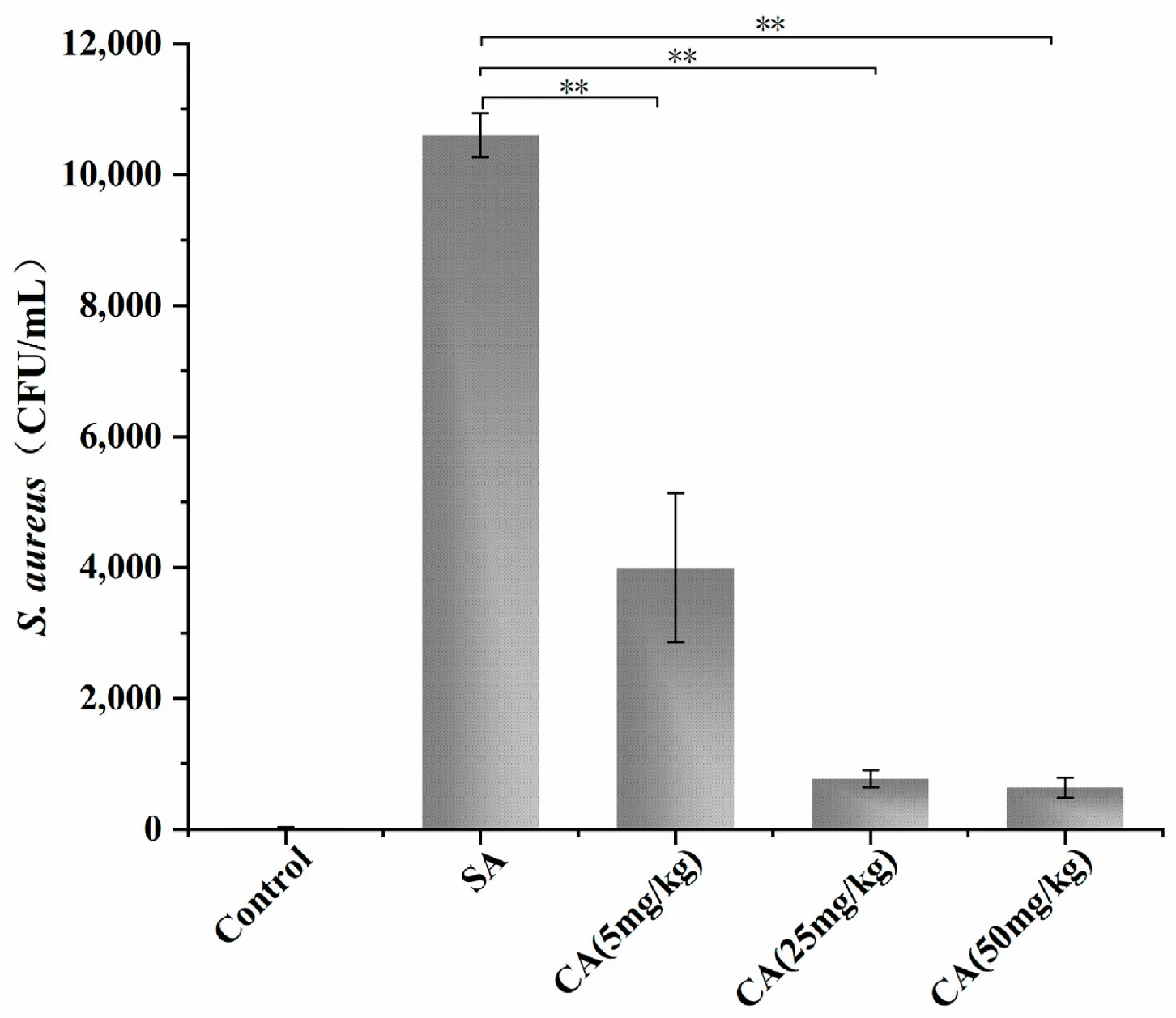
The effect of CA on the colonization of *S. aureus* in mouse mastitis. Note: ** means extremely significant difference (*p* < 0.01), *n* = 7 mice per group.

To further investigate the effect of CA when alleviating the mammary inflammatory response in mice, the impact of *S. aureus* colonization in mouse mammary tissue was assessed using the colony counting method. The results showed that, compared with the control group, bacterial loads in the mammary glands of *S. aureus*-infected mice were significantly elevated. In contrast, *S. aureus* colonization in the CA treated groups was markedly reduced at different doses (*n* = 7 mice per group, *p* < 0.01, Figure 3). These findings suggest that CA possesses potent antibacterial and anti-inflammatory effects, effectively inhibiting *S. aureus* colonization in mouse mammary tissue. Notably, a dose of 50 mg/kg demonstrated substantial therapeutic efficacy in alleviating mastitis. Overall, these results indicate that CA reduces MPO activity in mammary tissue, which in turn inhibits bacterial colonization and the mitigation of inflammatory injury in mouse mammary glands.

### 3.4. Effect of CA on Oxidative Stress Markers in Mouse Mastitis 

**Figure 4:**
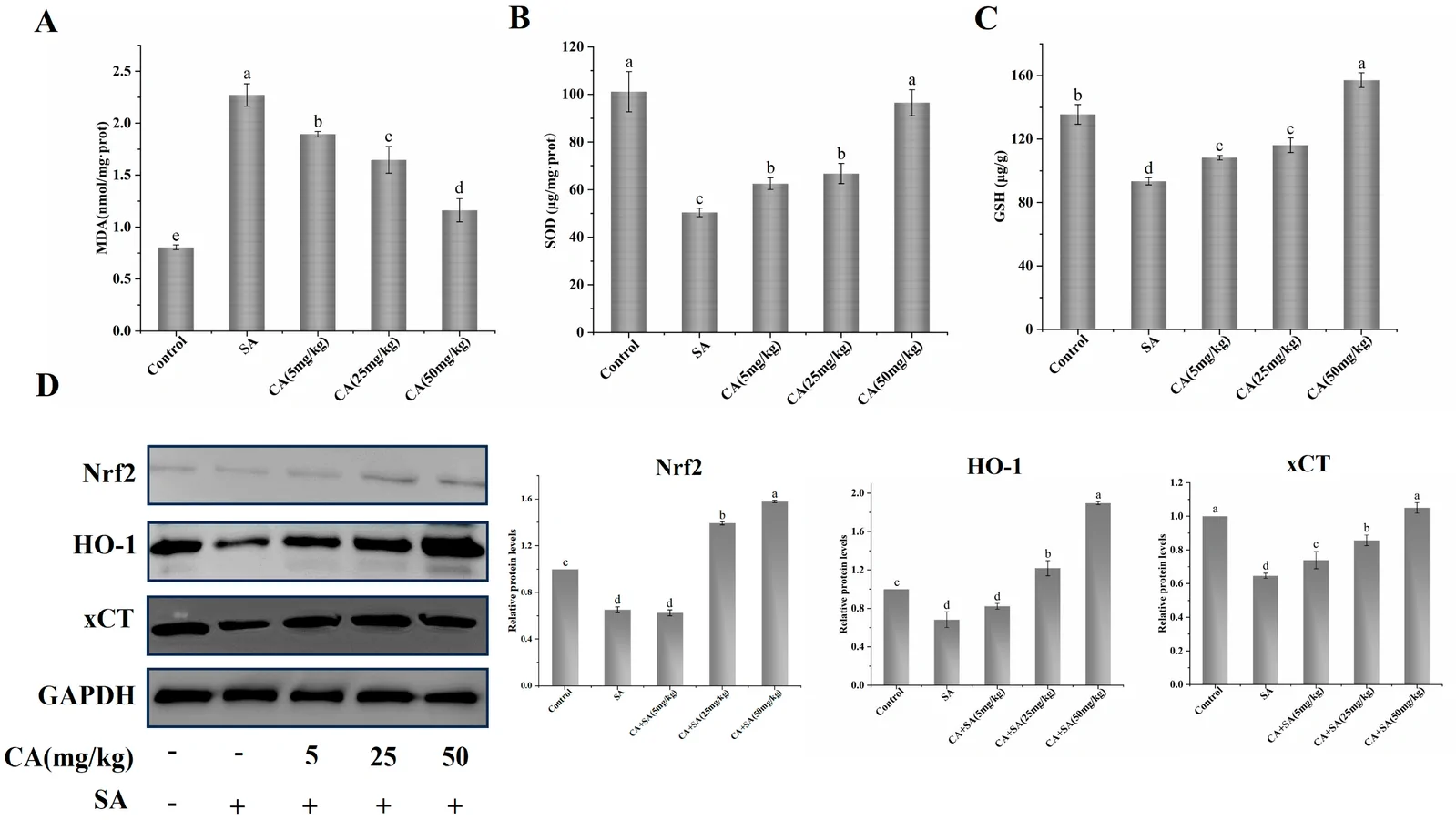
Impact of CA on oxidative stress markers in mouse mastitis. Note: (**A**) MDA levels, (**B**) SOD activity, (**C**) GSH-Px activity, (**D**) the effect of CA on the expression of Nrf2/HO-1/xCT in mouse mastitis, *n* = 7 mice per group. The significance of differences among groups is denoted by lowercase letters, where groups sharing the same letter are not significantly different, whereas groups with different letters indicate a statistically significant difference at *p* < 0.05.

Changes in antioxidant enzymes, free radicals, and oxidation products can effectively reflect the degree of oxidative stress injury in the organism. MDA is one of the final products of lipid peroxidation, SOD is a major scavenger of oxygen free radicals, and GSH-Px represents the overall peroxidase activity. To further investigate the effect of CA on oxidative stress in mouse mastitis, we measured SOD and GSH-Px activities and MDA levels in mammary gland tissues using commercial ELISA kits. The results showed that, compared with the control group, MDA levels in the mammary tissue of *S. aureus*-infected mice were significantly elevated, whereas the activities of SOD and GSH-Px were markedly decreased. Treatment with different concentrations of CA ameliorated these alterations. Specifically, MDA levels were significantly reduced, while SOD and GSH-Px activities were significantly increased relative to the *S. aureus* infection group (*n* = 7 mice per group, *p* < 0.05, Figure 4A–C).

To further clarify the mechanism by which CA alleviates oxidative stress-induced damage in mouse mammary tissue, Western blot analysis was performed to evaluate the effect of CA on the Nrf2/HO-1/xCT antioxidant pathway in mouse model of mastitis. The results revealed that the expression levels of Nrf2 and its downstream proteins, HO-1 and xCT, were markedly reduced in the mammary glands of *S. aureus*-infected mice. In contrast, CA treatment significantly upregulated the expression of Nrf2, HO-1, and xCT in a dose-dependent manner (*n* = 7 mice per group, *p* < 0.05, Figure 4D). These findings suggest that CA alleviates oxidative stress-related damage induced by *S. aureus* infection by activating the Nrf2/HO-1/xCT antioxidant pathway, thereby lowering MDA levels, promoting GSH-Px production, and increasing SOD activity.

### 3.5. Effect of CA on the Inflammatory Response in Mouse Mastitis

**Figure 5:**
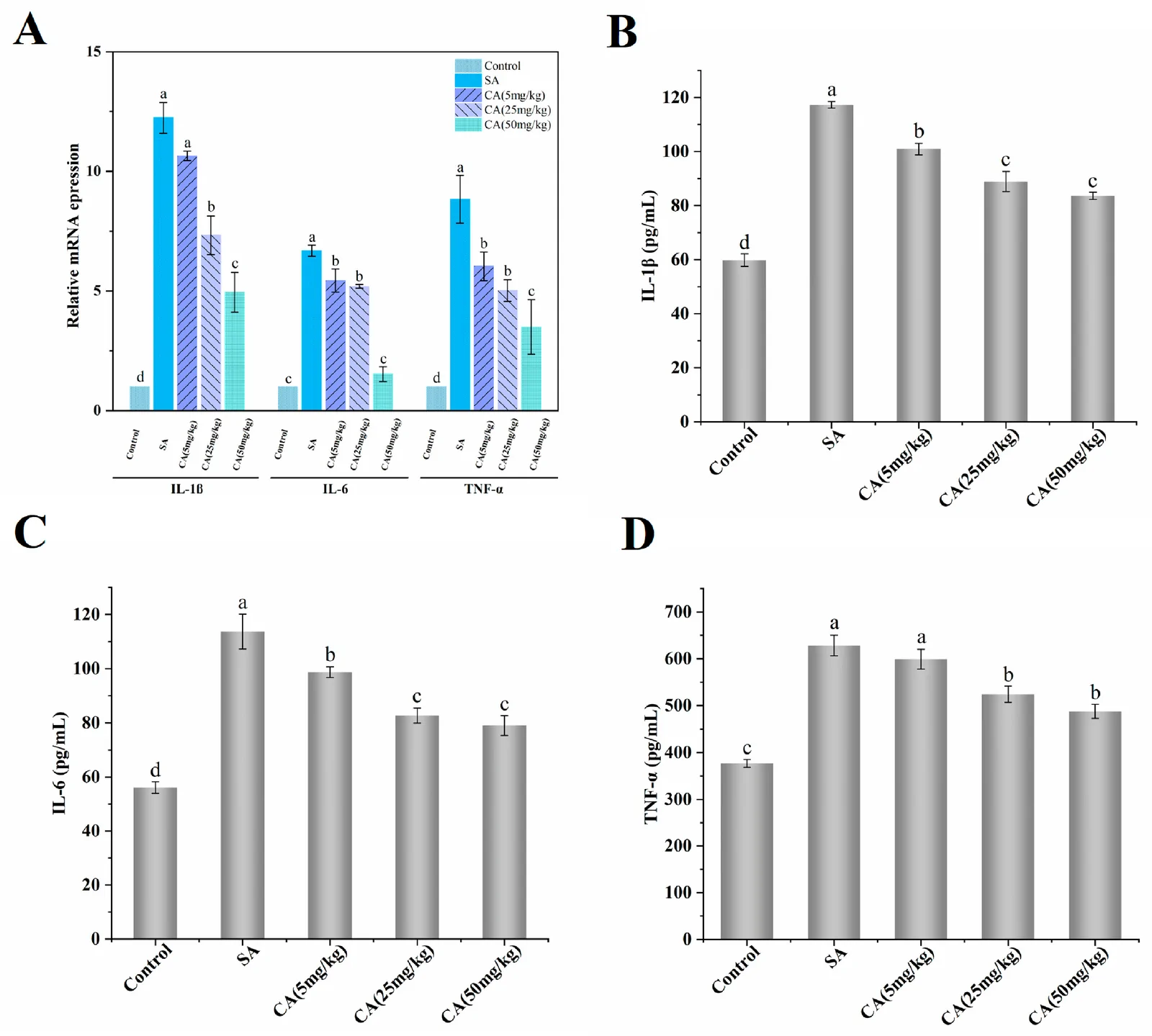
Effect of CA on the expression of inflammatory factors in mouse mastitis. Note: (**A**) RT-qPCR was used to quantify the mRNA expression levels of the inflammatory cytokines IL-1β, IL-6, and TNF-α in mouse mastitis, (**B**) serum IL-1β levels were quantified by ELISA, (**C**) serum IL-6 levels were quantified by ELISA, (**D**) serum TNF-α levels were quantified by ELISA, *n* = 7 mice per group. The significance of differences among groups is denoted by lowercase letters, where groups sharing the same letter are not significantly different, whereas groups with different letters indicate a statistically significant difference at *p* < 0.05.

To further investigate the impact of CA on the inflammatory response in the mouse mammary gland, RT-qPCR and ELISA were utilized to quantify the levels of pro-inflammatory cytokines. RT-qPCR analysis revealed that the expression of IL-1β, IL-6, and TNF-α in the mammary gland tissues of *S. aureus*-infected mice was markedly increased compared with the control group. In contrast, CA treatment substantially reduced the expression of TNF-α, IL-1β, and IL-6 in the mammary glands of infected mice (*n* = 7 mice per group, *p* < 0.05, Figure 5A). Consistently, ELISA results showed that CA markedly lowered the serum concentrations of TNF-α, IL-1β, and IL-6 (*n* = 7 mice per group, *p* < 0.05, Figure 5B–D), aligning with the RT-qPCR findings.

### 3.6. Effect of CA on the Expression of TLR4/NF-κB Pathway in Mouse Mastitis

**Figure 6:**
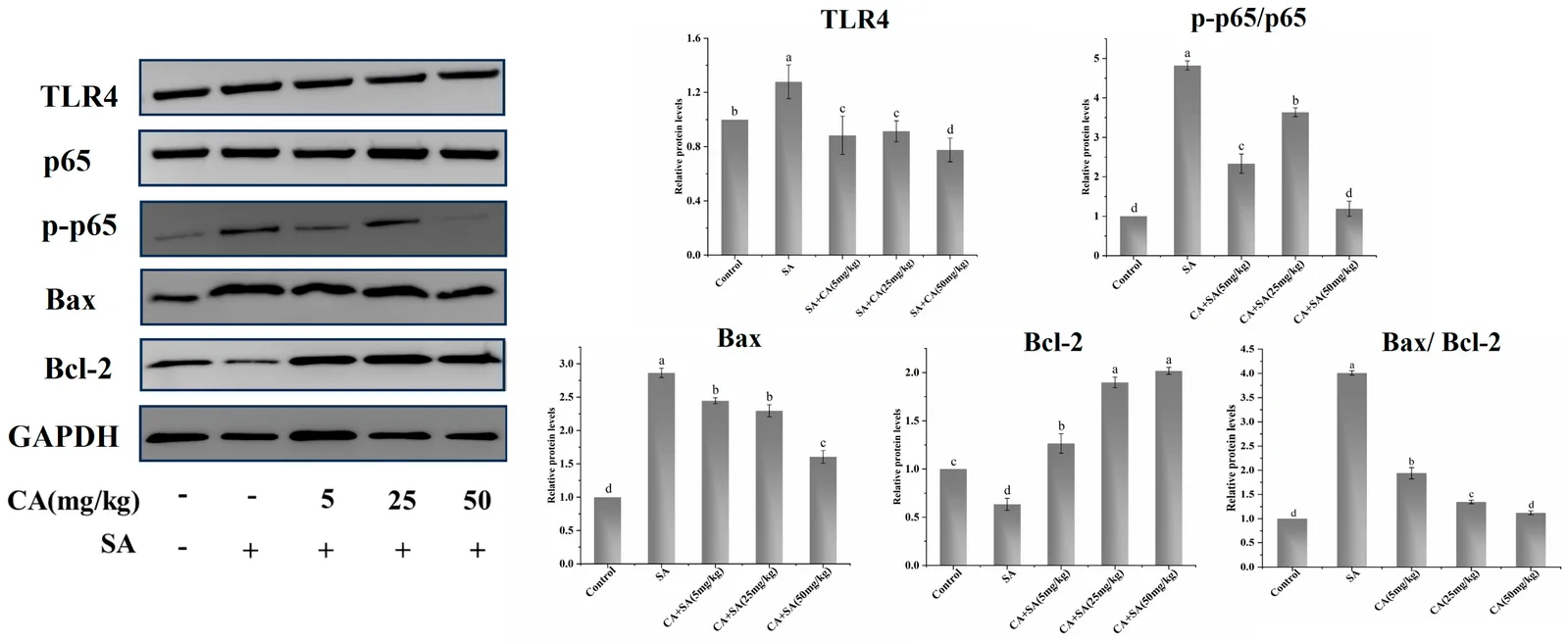
The effect of CA on the expression of TLR4/NF-κB in mouse mastitis. Note: The significance of differences among groups is denoted by lowercase letters, where groups sharing the same letter are not significantly different, whereas groups with different letters indicate a statistically significant difference at *p* < 0.05.

To clarify the protein-level alterations involved in CA-mediated attenuation of inflammatory injury in mouse mammary tissue, Western blot analysis was conducted to quantify TLR4, p65, p-p65, Bax, and Bcl-2. The results revealed that *S. aureus* infection markedly increased TLR4 expression, enhanced p-p65 phosphorylation, and significantly elevated the Bax/Bcl-2 ratio when compared with the control group, indicating activation of the TLR4/NF-κB inflammatory pathway. In contrast, treatment with increasing doses of CA markedly attenuated these changes, as evidenced by reduced TLR4 expression, decreased p-p65 phosphorylation, and a lowered Bax/Bcl-2 ratio in a dose-dependent manner (*n* = 7 mice per group, *p* < 0.05, Figure 6). These findings suggest that CA mitigates the inflammatory response in the mammary glands of mice by suppressing activation of the TLR4/NF-κB pathway and inhibiting apoptosis.

### 3.7. Effect of CA on the Viability of MAC-T Cells and the Expression of Inflammatory Factors

**Figure 7:**
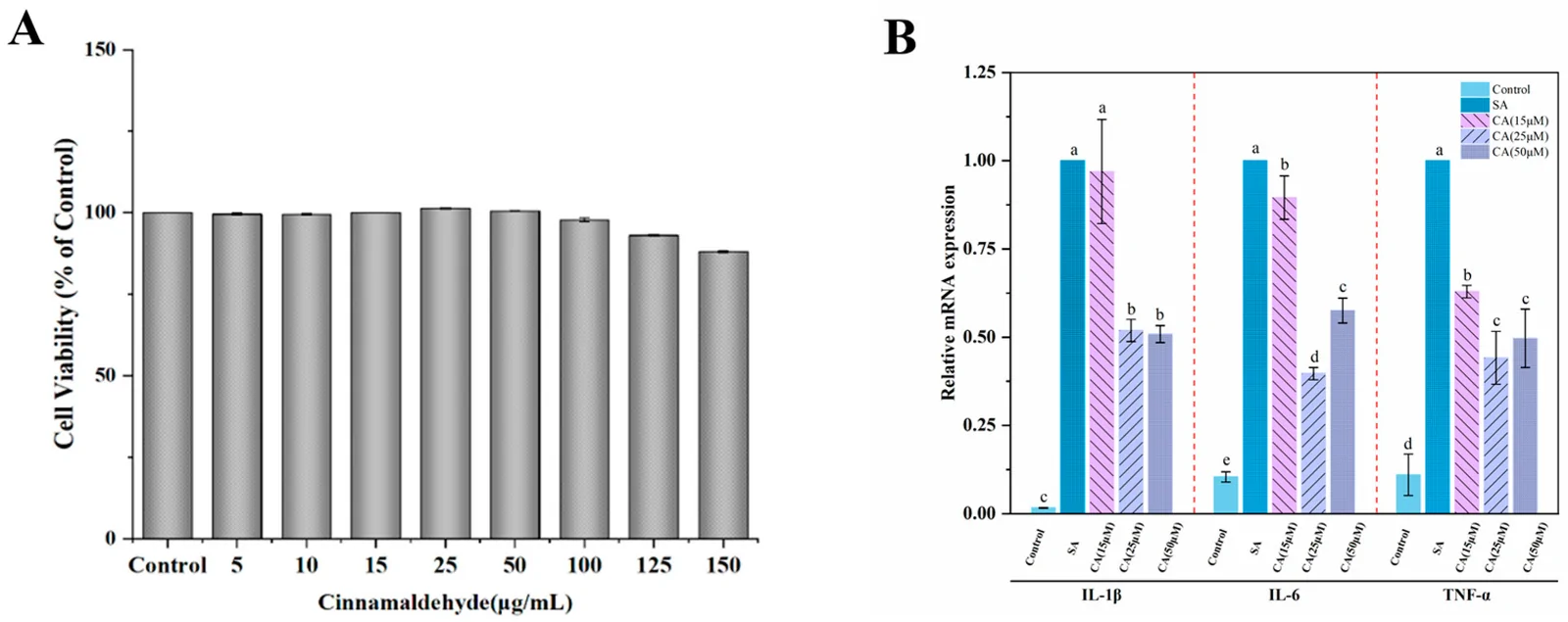
The effect of CA on the activity of MAC-T cells and the expression of inflammatory factors. Note: (**A**) the effect of CA on the viability of MAC-T cells, (**B**) the effect of CA on the expression of inflammatory factors, *n* = 3 independent experiments. The significance of differences among groups is denoted by lowercase letters, where groups sharing the same letter are not significantly different, whereas groups with different letters indicate a statistically significant difference at *p* < 0.05.

To determine the optimal concentration range of CA for MAC-T cells in vitro, a CCK-8 assay was employed to evaluate the effect of CA concentration on cell viability. The results showed that at a concentration below 50 μM, MAC-T cell viability was not significantly affected. However, at a concentration exceeding 50 μM, MAC-T cell viability decreased (*n* = 3 independent experiments, Figure 7A). 

Additionally, the expression of inflammatory factors IL-1β, IL-6, and TNF-α in MAC-T cells was measured by RT-qPCR to evaluate the impact of CA on *S. aureus*-induced inflammatory responses. The results indicated that, compared with the control group, the mRNA expression levels of IL-1β, IL-6, and TNF-α were significantly elevated following *S. aureus* infection (*n* = 3 independent experiments, *p* < 0.05). After pretreatment with 15, 25 or 50 μM CA, the mRNA levels of IL-1β, IL-6, and TNF-α were markedly reduced, with 25 μM CA showing the most pronounced inhibitory effect (Figure 7B). These findings suggest that CA mitigates *S. aureus*-induced inflammatory responses in MAC-T cells by suppressing the expression of inflammatory factors. 

### 3.8. Effect of CA on the Invasion and Adhesion in MAC-T Cells

**Figure 8:**
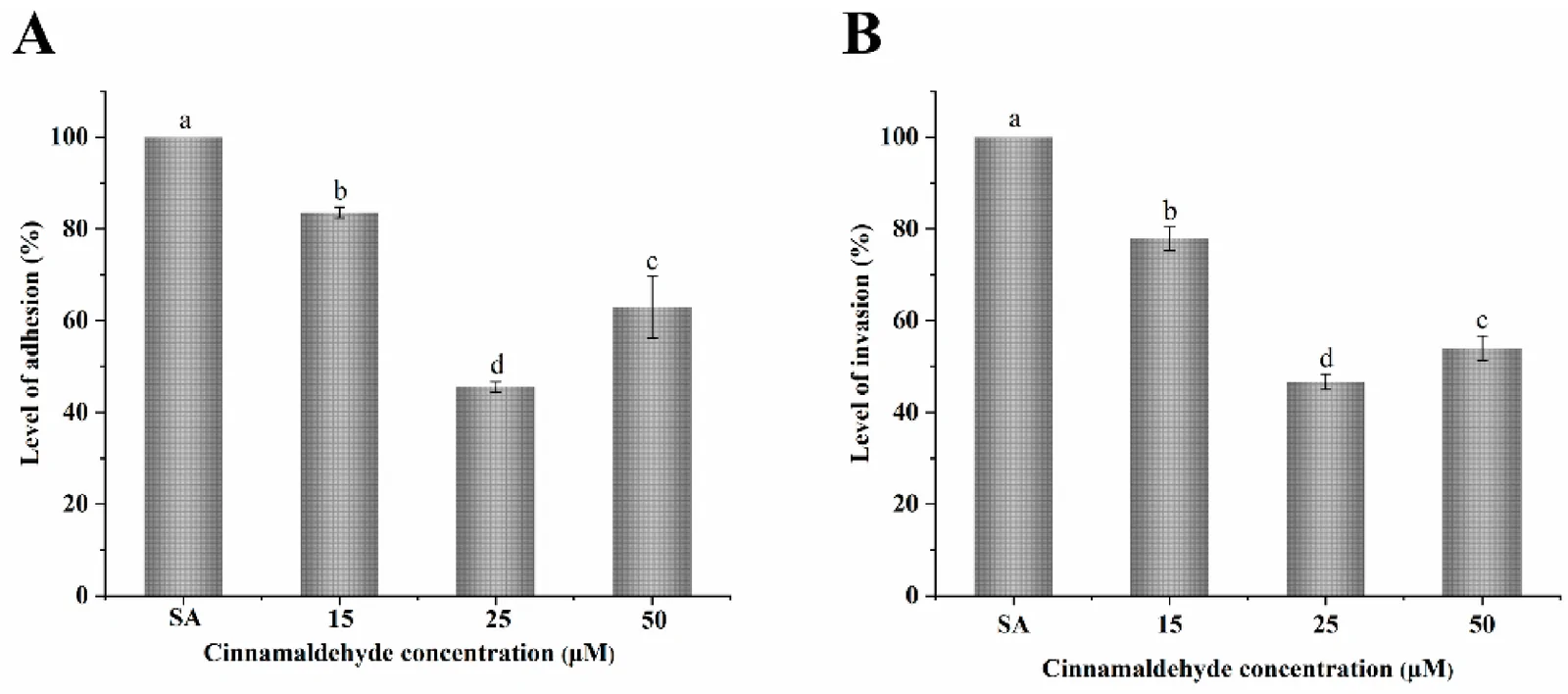
The effect of CA on invasion and adhesion of *S. aureus* in MAC-T cells. Note: (**A**) the adhesion of *S. aureus* in MAC-T cells, (**B**) the invasion of *S. aureus* in MAC-T cells, *n* = 3 independent experiments. The significance of differences among groups is denoted by lowercase letters, where groups sharing the same letter are not significantly different, whereas groups with different letters indicate a statis-tically significant difference at *p* < 0.05.

In this study, the effect of CA on the adhesion and invasion of *S. aureus* in MAC-T cells was assessed using a plate colony counting assay. The results demonstrated that, compared with the control group, the adhesion and invasion rates of *S. aureus* in MAC-T cells were significantly reduced after pretreatment with 15, 25 or 50 μM CA. Specifically, the adhesion rates were reduced to 83.6%, 45.57%, and 62.9%, respectively (*n* = 3 independent experiments, *p* < 0.05, Figure 8A). In the invasion assay, the invasion rate of *S. aureus* to MAC-T cells was reduced to 77.9% after pretreatment with 15 μM CA, and to 46.6% and 54.0% following pretreatment with 25 and 50 μM CA, respectively (*n* = 3 independent experiments, *p* < 0.05, Figure 8B). These results suggest that CA at the tested concentrations significantly inhibits *S. aureus* adhesion and invasion in MAC-T cells, which may contribute to mitigating the *S. aureus*-induced inflammatory response.

### 3.9. Effect of CA on Oxidative Stress in MAC-T Cells

**Figure 9:**
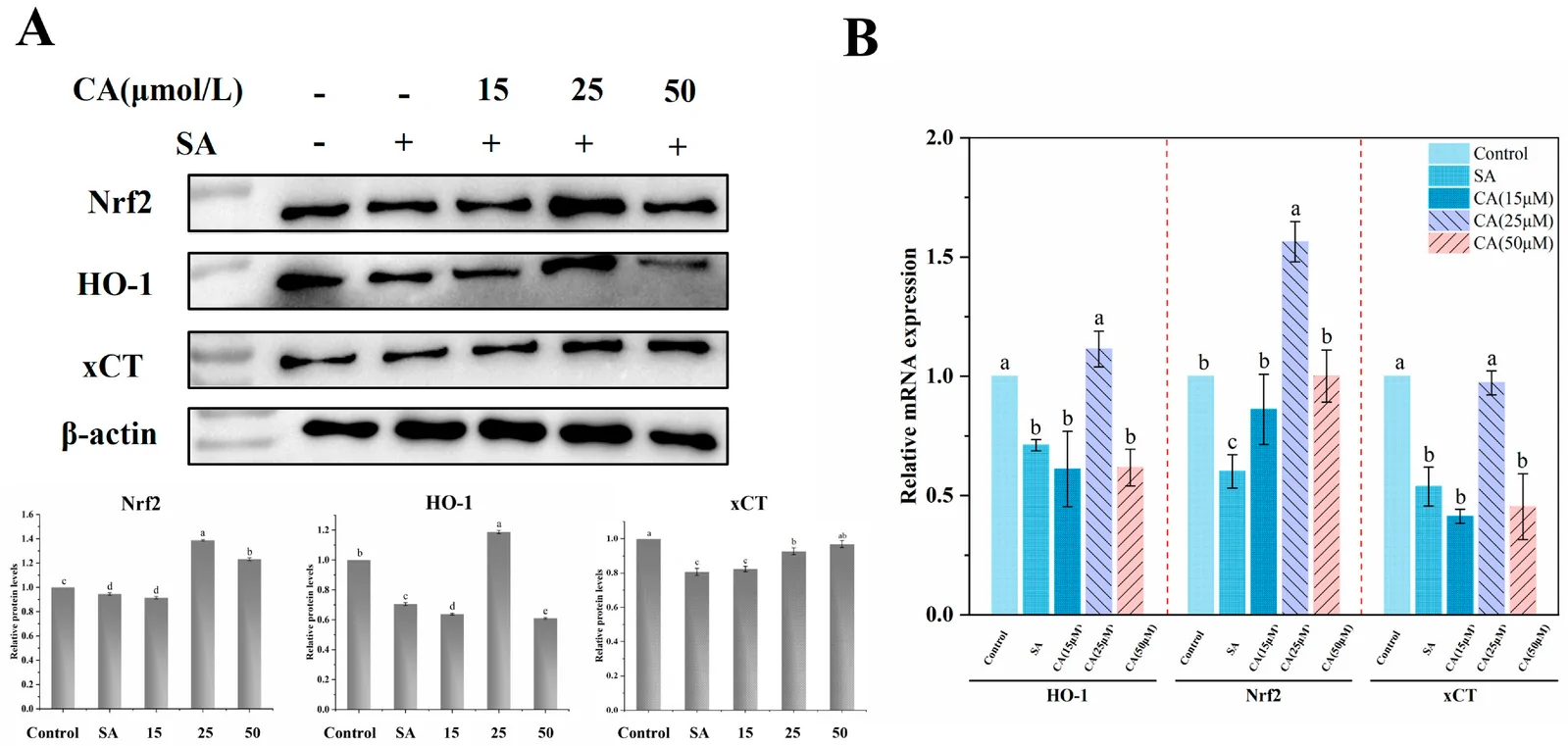
The effect of CA on Nrf2/HO-1/xCT pathway in MAC-T cells. Note: (**A**) the effect of CA on the expression of Nrf2/HO-1/xCT protein, (**B**) the effect of CA on the mRNA expression levels of *Nrf2*, *HO-1*, and *xCT* genes, *n* = 3 independent experiments. The significance of differences among groups is denoted by lowercase letters, where groups sharing the same letter are not significantly different, whereas groups with different letters indicate a statistically significant difference at *p* < 0.05.

Similarly, to investigate the effect of CA on the Nrf2/HO-1/xCT pathway in *S. aureus*-induced MAC-T cells in vitro, we performed RT-qPCR and Western blot analyses to evaluate the expression of related genes and proteins. Western blot analysis revealed that CA significantly increased the expression levels of Nrf2, HO-1, and xCT proteins in *S. aureus*-infected MAC-T cells (*n* = 3 independent experiments, *p* < 0.05, Figure 9A). RT-qPCR analysis further demonstrated that CA significantly upregulated the mRNA expression levels of *Nrf2*, *HO-1*, and *xCT* genes (*n* = 3 independent experiments, *p* < 0.05, Figure 9B). These results suggest that CA alleviates oxidative stress-induced damage in *S. aureus*-induced MAC-T cells by activating the Nrf2/HO-1/xCT antioxidant pathway.

**Figure 10:**
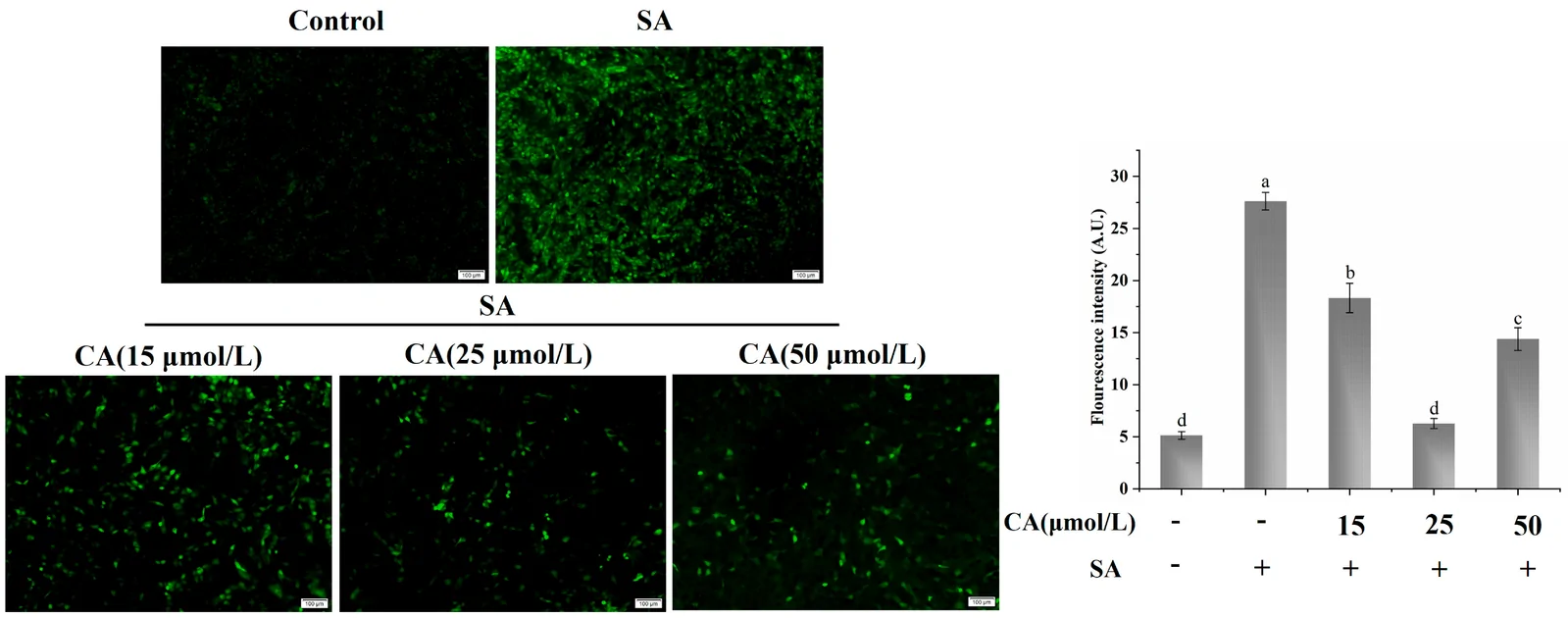
The effect of CA on the production of active ROS in MAC-T cells. Note: The significance of differences among groups is denoted by lowercase letters, where groups sharing the same letter are not significantly different, whereas groups with different letters indicate a statistically significant difference at *p* < 0.05.

Intracellular ROS play a pivotal role in the cellular inflammatory response. In this study, we evaluated the effect of CA on the production of active ROS in *S. aureus*-infected MAC-T cells using fluorescent dye staining. The results revealed that, compared with the control group, ROS production was significantly increased in *S. aureus*-infected MAC-T cells (*p* < 0.05). Pretreatment with 15, 25 or 50 μM CA significantly inhibited the *S. aureus*-induced intracellular ROS accumulation (*n* = 3 independent experiments, *p* < 0.05, Figure 10). These findings indicate that CA effectively attenuates *S. aureus*-induced ROS accumulation in MAC-T cells.

### 3.10. Effect of CA on the Expression of Inflammatory Mediators and Tight Junction Proteins in MAC-T Cells

**Figure 11:**
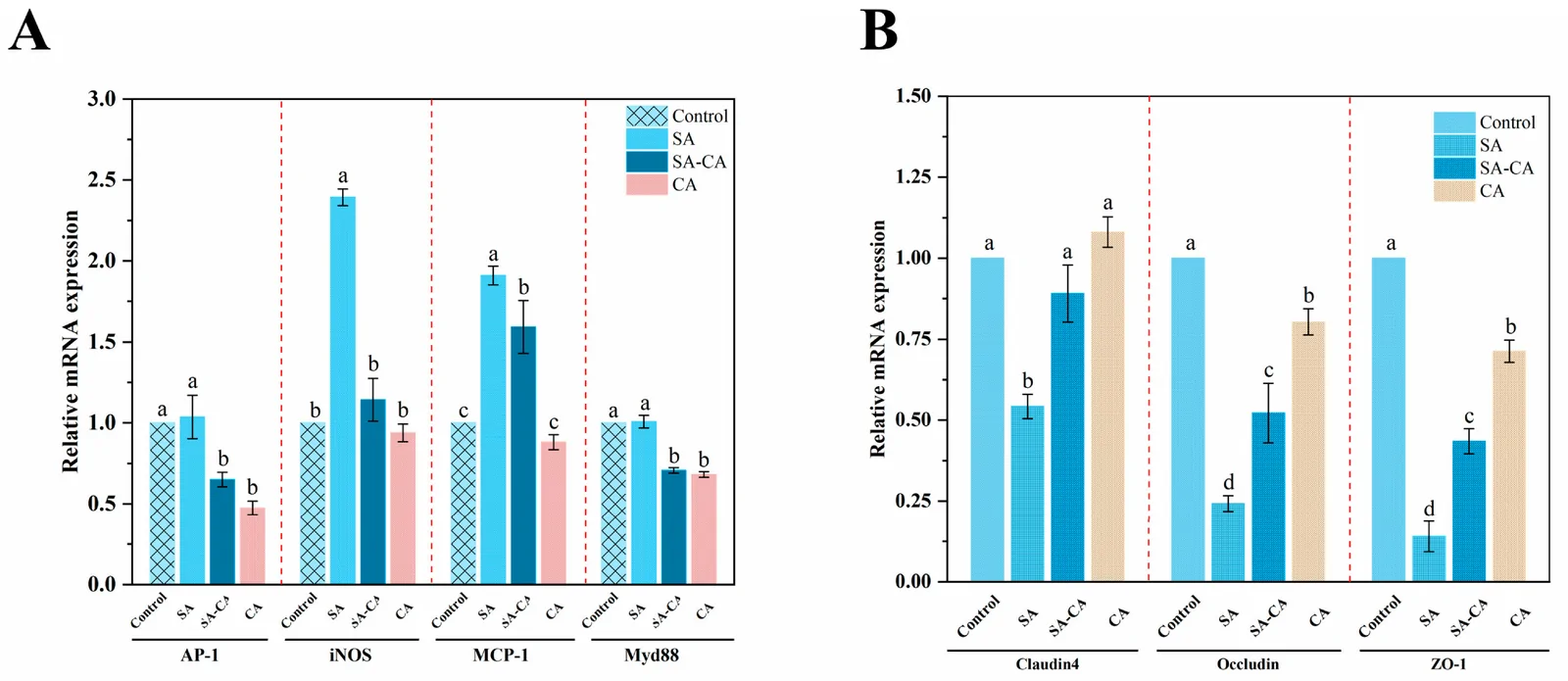
The effect of CA on the expression of inflammatory mediators and tight junction proteins. Note: (**A**) the effect of CA on the expression of inflammatory mediators in MAC-T cells, (**B**) the effect of CA on the expression of tight junction proteins in MAC-T cells, *n* = 3 independent experiments. The significance of differences among groups is denoted by lowercase letters, where groups sharing the same letter are not significantly different, whereas groups with different letters indicate a statis-tically significant difference at *p* < 0.05.

This research further analysed the effects of CA on the expression of inflammatory mediators (AP-1, iNOS, MCP-1, and Myd88) and tight junction proteins (occludin, claudin 4, and ZO-1) in *S. aureus*-induced MAC-T cells using RT-qPCR. Compared with the *S. aureus*-induced group, CA significantly downregulated the mRNA expression levels of AP-1, iNOS, MCP-1, and Myd88 in MAC-T cells (*n* = 3 independent experiments, *p* < 0.05, Figure 11A). *S. aureus* infection significantly reduced the mRNA expression of the tight junction proteins occludin, claudin 4, and ZO-1 (*p* < 0.05). However, CA pretreatment significantly upregulated the mRNA expression of these tight junction proteins (*n* = 3 independent experiments, *p* < 0.05, Figure 11B). These findings indicate that CA exerts anti-inflammatory effects by suppressing inflammatory mediator expression and promoting tight junction protein expression in MAC-T cells, thereby preserving mammary epithelial barrier integrity.

### 3.11. Effect of CA on Inflammatory Response of MAC-T Cells

**Figure 12:**
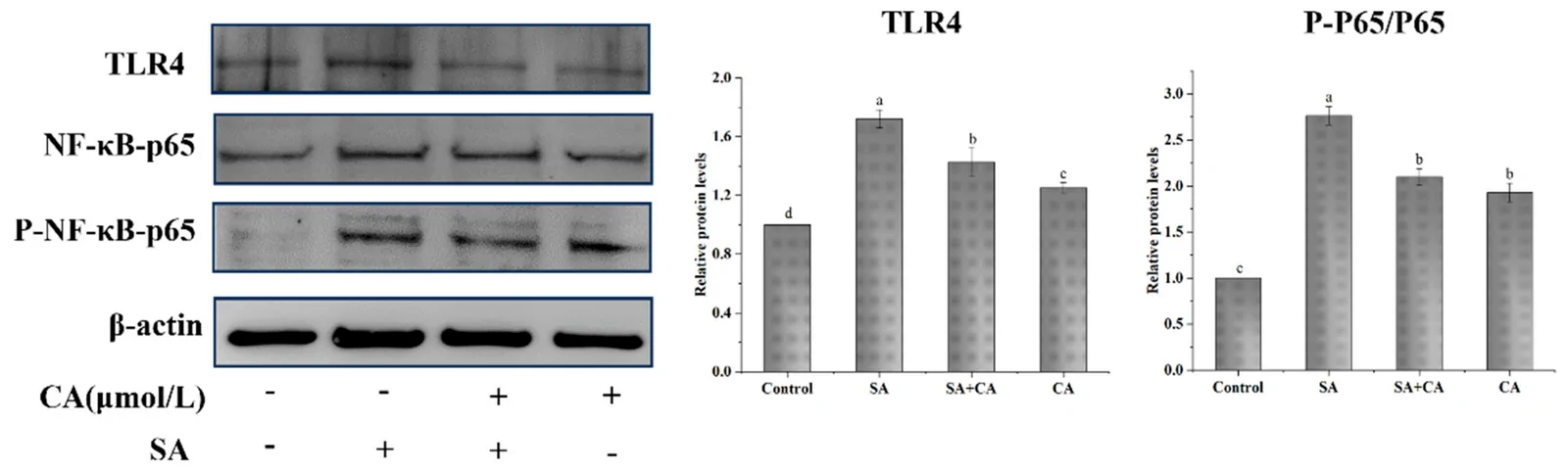
The effect of CA on *S. aureus*-induced TLR4/NF-κB pathway in MAC-T cells. Note: The significance of differences among groups is denoted by lowercase letters, where groups sharing the same letter are not significantly different, whereas groups with different letters indicate a statis-tically significant difference at *p* < 0.05.

Western blot analysis further revealed that CA significantly downregulated *S. aureus*-induced TLR4 protein expression and p65 phosphorylation in MAC-T cells, indicating inhibition of the TLR4/NF-κB inflammatory pathway and reduction of inflammatory mediator expression (*n* = 3 independent experiments, *p* < 0.05, Figure 12).

### 3.12. Effect of CA on S. aureus-Induced Apoptosis in MAC-T Cells

**Figure 13:**
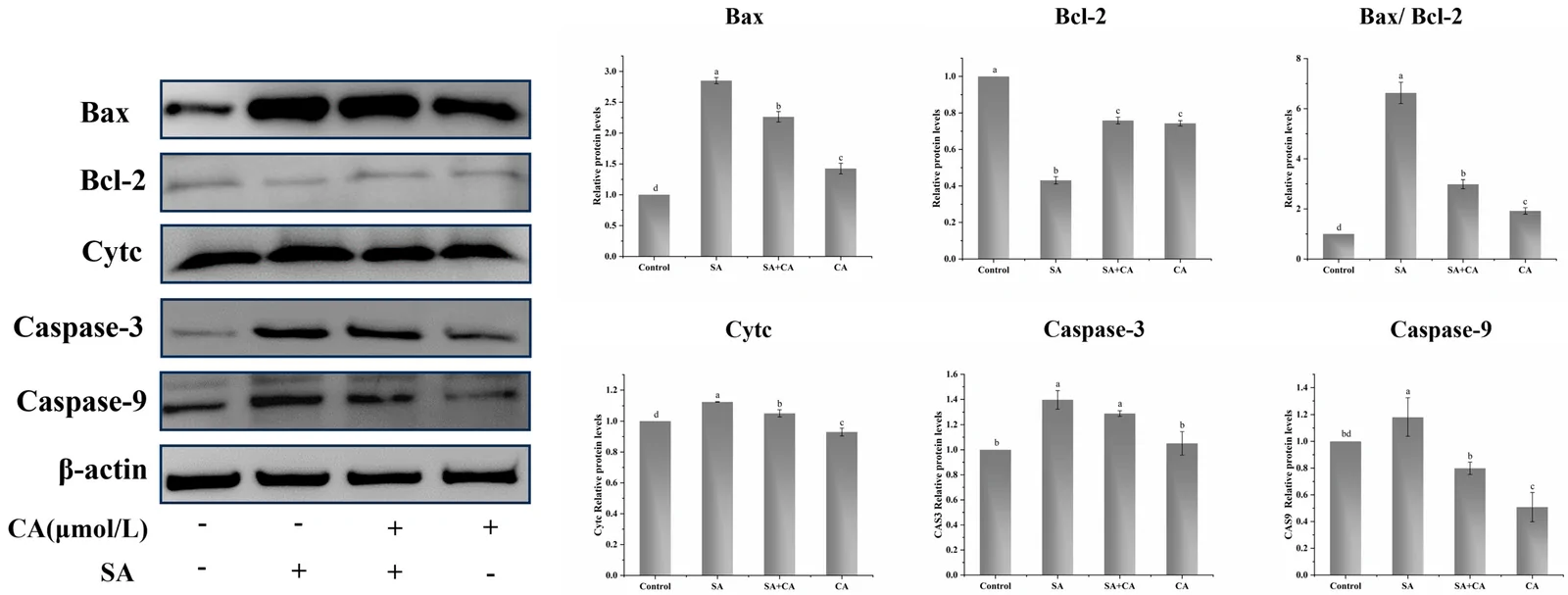
The effect of CA on the apoptosis of MAC-T cells induced by *S. aureus.* Note: The significance of differences among groups is denoted by lowercase letters, where groups sharing the same letter are not significantly different, whereas groups with different letters indicate a statistically significant difference at *p* < 0.05.

Bax, Bcl-2, and caspase-3 are well-recognized key regulators of apoptosis. Bax, a pro-apoptotic protein, induces apoptosis through caspase-3 activation, whereas Bcl-2, an anti-apoptotic protein, inhibits caspase-3 activation and plays a critical role in apoptosis regulation. Apoptosis is also characterized by Cytc release and enhanced caspase-9 activity. To further examine the effect of CA on apoptosis in MAC-T cells subjected to *S. aureus*-induced oxidative stress and inflammation, we assessed the expression of apoptotic proteins, including Bax, Bcl-2, caspase-3, caspase-9, and Cytc, by Western blot analysis in vitro. Compared with the control and single-drug groups, *S. aureus* infection significantly upregulated Bax, caspase-3, caspase-9, and Cytc expression, whereas Bcl-2 expression was markedly downregulated in MAC-T cells. CA pretreatment reversed these alterations (*n* = 3 independent experiments, Figure 13).

## 4. Discussion

Inflammation refers to the complex immune response to various pathogens, toxic compounds, and environmental factors; however, excessive or prolonged inflammatory responses may adversely affect the host [24]. If the inflammatory response is not promptly resolved, it may result in the development of pathological conditions [25]. Moreover, insufficient treatment of inflammation or persistent pathogen-induced stimulation may cause continuous immune cell activation and sustained inflammatory responses [26]. Numerous studies have demonstrated that plant extracts rich in natural bioactive compounds can effectively attenuate inflammatory responses, underscoring their potential as therapeutic agents for inflammatory diseases. 

Toll-like receptors (TLRs) function as pattern recognition receptors (PRRs) in the innate immune system. Among the TLR family, TLR4 has been extensively reported as a key receptor implicated in inflammatory diseases [27]. TLR4 can activate the NF-κB signalling pathway during acute inflammatory responses and apoptosis, resulting in the nuclear accumulation of phosphorylated p65 (p-p65) and the subsequent induction of inflammatory responses through the secretion of pro-inflammatory cytokines, including TNF-α, IL-6, and IL-1β [28,29]. Thus, inhibition of NF-κB signalling has emerged as a promising strategy for controlling inflammation [30]. In the mammary gland, inflammation is accompanied by the production of various cytokines, including pro- and anti-inflammatory mediators, which orchestrate the inflammatory process. TNF-α and IL-1β play essential roles in mediating leukocyte recruitment to inflammatory sites [31]. As a major mediator of endotoxin-induced toxicity, TNF-α is among the initial cytokines triggering excessive inflammatory responses. Furthermore, TNF-α collaborates with other cytokines, including interleukins and interferons, to regulate inflammatory processes, these pro-inflammatory mediators can further enhance the growth and proliferation of *S. aureus* in bovine mammary epithelial cells [32]. It has been demonstrated that CA mitigates *Cronobacter sakazakii* (*C. sakazakii*)-induced intestinal inflammation in mice through the downregulation of IL-1β, IL-6, and TNF-α expression in intestinal epithelial cells, suppression of NF-κB pathway activation, and reduction of Caspase-3 activity [33]. Furthermore, recent research has shown that CA reduces *Salmonella* colonization in the mouse intestine and enhances intestinal barrier function by inhibiting the expression of the effector protein encoded by salmonella pathogenicity island 1 (SPI-1) [34]. In addition, CA has been reported to mitigate lipopolysaccharide (LPS)-induced macrophage immune responses [35]. Collectively, these findings highlight CA as a potential therapeutic candidate for inflammatory disease treatment. In this study, the expression levels of TNF-α, IL-1β, and IL-6 in mammary tissues and serum were measured using RT-qPCR and ELISA. CA treatment significantly decreased TNF-α, IL-1β, and IL-6 levels in a dose-dependent manner. The 50 mg/kg CA treatment significantly reduced MPO activity and *S. aureus* colonization, and mitigated inflammatory cell infiltration in mouse mastitis, which was consistent with the decreased expression of inflammatory mediators. Furthermore, CA inhibited TLR4/NF-κB pathway activation and downregulated apoptotic protein expression by decreasing TLR4 and p-p65 phosphorylation, thereby alleviating inflammatory damage in mouse mastitis. Similarly, 25 μM CA attenuated *S. aureus*-induced inflammatory damage in MAC-T cells in vitro by targeting the TLR4/NF-κB pathway, which suppressed the expression of inflammatory mediators and concurrently upregulated the mRNA levels of tight junction proteins. These findings demonstrate that CA has a favourable effect in alleviating mammary inflammation. CA can act directly on mammary epithelial cells to modulate the host cell inflammatory response. Together, these results lay the groundwork for the clinical application of cinnamon essential oil in the alleviation and treatment of bovine mastitis.

Nrf2 plays a central role in cellular defence against oxidative damage and inflammation by regulating more than 250 genes involved in antioxidant and anti-inflammatory responses [36,37]. Upon oxidative stress, including elevated intracellular ROS levels, Nrf2 becomes activated and translocates to the nucleus, where it modulates the expression of downstream antioxidant genes such as HO-1 and xCT. This activation enhances the expression of antioxidant enzymes, including GSH-Px, SOD, thereby mitigating oxidative damage and maintaining intracellular redox homeostasis [38]. HO-1, a key antioxidant enzyme, is generally expressed at low levels under physiological conditions. However, under oxidative stress, HO-1 expression is upregulated to protect cells from damage. xCT, a cystine transporter, contributes to the regulation of GSH synthesis [39]. CA is additionally recognized as an activator of Nrf2, a critical transcription factor that mitigates oxidative stress, endothelial dysfunction, and inflammation associated with metabolic disorders [40]. To evaluate the effect of CA on oxidative stress in mastitis, this study measured MDA levels and SOD and GSH-Px activities in a mouse model of mastitis *in vivo*. The results showed that the 50 mg/kg CA treatment significantly decreased MDA level in mastitis tissue, enhanced SOD and GSH-Px activities, and upregulated Nrf2 and its downstream antioxidant enzymes, HO-1 and xCT, thereby activating the Nrf2/HO-1/xCT pathway and mitigating oxidative stress in the mammary glands of mice. Meanwhile, results from the in vitro MAC-T cell model showed that 25 μM CA alleviated oxidative stress by activating the Nrf2/HO-1/xCT pathway and reducing ROS accumulation.

ROS accumulation is closely linked to oxidative stress and inflammatory responses. Previous studies have shown that *S. aureus*-induced oxidative stress in mammary epithelial cells leads to ROS accumulation and triggers intracellular immune responses [41]. Therefore, inhibiting ROS accumulation represents an established approach for alleviating inflammatory diseases, such as mastitis. In this study, we evaluated the effect of CA on the production of active ROS in *S. aureus*-infected MAC-T cells using fluorescent dye staining. The results revealed that ROS production was significantly increased in *S. aureus*-infected MAC-T cells and that CA significantly inhibited the *S. aureus*-induced intracellular ROS accumulation. These findings indicate that CA effectively attenuates *S. aureus*-induced ROS accumulation in MAC-T cells.

In the present study, employing a mouse mastitis model *in vivo* and a MAC-T cell inflammatory model *in vitro*, the mechanism of action of CA was preliminarily explored. The results indicated that CA decreased the levels of inflammatory cytokines and mediators, suppressed TLR4/NF-κB pathway activation, reduced intracellular ROS accumulation, activated the Nrf2/HO-1/xCT antioxidant pathway, and downregulated apoptotic protein expression, suggesting that CA can directly modulate host cell inflammatory and oxidative stress responses. Furthermore, in the in vivo mouse mastitis model, a marked reduction in *S. aureus* colonization in mammary tissues was observed, which may also contribute to the alleviation of inflammation by limiting PAMPs. Thus, CA appears to exert its anti-inflammatory effects through a dual mechanism: direct modulation of host immune signalling and indirect reduction of bacterial burden. Nevertheless, further studies are warranted to identify the specific immune receptors with which CA interacts. In summary, this study confirms that CA effectively alleviates *S. aureus*-induced mammary inflammation both *in vitro* and *in vivo*. Optimal efficacy was observed at 25 μM in MAC-T cells *in vitro* and at 50 mg/kg in a mouse model of mastitis *in vivo*, both within safe concentration/dose ranges. These results suggest that CA holds promise as a natural anti-mastitis candidate and may offer a new alternative to antibiotic-based strategies. However, this study was validated only in a mouse model. In future work, we will further investigate the pharmacokinetics and long-term safety of CA, as well as determine the optimal dosing regimen and efficacy in a bovine mastitis model, thereby laying a foundation for the clinical application of cinnamon essential oil.

## 5. Conclusions

CA markedly inhibited *S. aureus* colonization in mammary tissue by mitigating inflammatory cell infiltration, preserving glandular structure, downregulating pro-inflammatory cytokines and apoptotic proteins, and reducing MPO activity. Furthermore, CA suppressed TLR4/NF-κB pathway activation, thereby mitigating inflammation in infected mammary glands. CA further alleviated oxidative stress by decreasing MDA levels, enhancing SOD and GSH-Px activities, and activating the Nrf2/HO-1/xCT antioxidant pathway. In the *in vitro* infection model, CA inhibited *S. aureus* adhesion and invasion in MAC-T cells, downregulated inflammatory cytokines and mediators, reduced intracellular ROS accumulation, and activated the Nrf2/HO-1/xCT signalling pathway. 

## Figures and Tables

**Table 1:** Sequences of RT-qPCR primers for inflammatory mediators in mouse mammary gland.

Primer Name	Sequences (5′-3′)	Length (bp)
*GAPDH*_F	GGAGCGAGACCCCACTAACAT	138
*GAPDH*_R	TAAGGGGGCTAAGCAGTTGGT	
*IL-1β*_F	GCTGCTTCCAAACCTTTGAC	206
*IL-1β*_R	AGCTTCTCCACAGCCACAAT	
*IL-6*_F	CCGGAGAGGAGACTTCACAG	363
*IL-6*_R	CAGAATTGCCATTGCACAAC	
*TNF-α*_F	ACGGCATGGATCTCAAAGAC	227
*TNF-α*_R	GTGGGTGAGGAGCACGTAGT	

**Table 2:** Sequences of RT-qPCR primers for cell adhesion molecules and inflammatory mediators.

Primer Name	Sequences (5′-3′)	Length (bp)
*GAPDH*_F	GGGTCATCATCTCTGCACCT	176
*GAPDH*_R	TAAGGGGGCTAAGCAGTTGGT	
*IL-1β*_F	GCTGCTTCCAAACCTTTGAC	209
*IL-1β*_R	AGAGGAGGTGGAGAGCCTTC	
*IL-6*_F	CACCCCAGGCAGACTACTTC	129
*IL-6*_R	TCCTTGCTGCTTTCACACTC	
*TNF-α*_F	GCCCTCTGGTTCAGACACTC	192
*TNF-α*_R	AGATGAGGTAAAGCCCGTCA	
*Nrf2_F*	CCAGCACAACACATACCATCAG	156
*Nrf2_R*	CGTAGCCGAAGAAACCTCATTG	
*HO-1_F*	GGCAGCAAGGTGCAAGA	221
*HO-1_R*	GAAGGAAGCCAGCCAAGAG	
*xCT_F*	GATACAAACGCCCAGATATGC	136
*xCT_R*	ATGATGAAGCCAATCCCTGTA	
*AP-1_F*	TCGTTCCTCCAGTCTGAGAGC	230
*AP-1_R*	GTCGGCGTGGTGGTGATATG	
*iNOS_F*	CTTGTTCTCGAGGTGCCCAT	174
*iNOS_R*	GTCCCGGACTCCAACTTCTG	
*MCP-1_F*	GTGCTCGCTCAGCCAGATGC	119
*MCP-1_R*	GGACACTTGCTGCTGGTGACTC	
*Myd88_F*	TCATTGAGAAGAGGTGCCGT	146
*Myd88_R*	TGGCTTGTACTTGATGGGGAT	
*Claudin 4_F*	CTTCATCGGCAGCAACATC	191
*Claudin 4_R*	ACAACAGCACGCCAAACA	
*Occludin_F*	GAACGAGAAGCGACTGTATC	122
*Occludin_R*	CACTGCTGCTGTAATGAGG	
*ZO-1_F*	TCTGCAGCAATAAAGCAGCATTTC	187
*ZO-1_R*	TTAGGGCACAGCATCGTATCACA	

## Data Availability

The data underlying this article will be shared on reasonable request to the corresponding author.

## Abbreviations

Cinnamaldehyde: CA; *Staphylococcus aureus*: *S. aureus*; dimethyl sulfoxide: DMSO; somatic cell counts: SCCs; antimicrobial resistance: AMR; phosphorylated p65: p-p65; *S. aureus* infection group: SA group; myeloperoxidase: MPO; malondialdehyde: MDA; superoxide dismutase: SOD; glutathione peroxidase: GSH-Px; pattern recognition receptors: PRRs; salmonella pathogenicity island 1: SPI-1; lipopolysaccharide: LPS; Mueller–Hinton agar: MHA; pathogen-associated molecular patterns: PAMPs; blood–milk barrier: BMB; bcl-2-associated X protein: Bax; B-cell lymphoma 2: Bcl-2; tumour necrosis factor-alpha: TNF-α; interleukin-1 beta: IL-1β; interleukin-6: IL-6; toll-like receptor 4: TLR4; nuclear factor kappa B: NF-κB; nuclear factor erythroid 2-related factor 2: Nrf2; heme oxygenase-1: HO-1; cystine/glutamate antiporter subunit: SLC7A11/xCT; reactive oxygen species: ROS. enzyme-linked immunosorbent assay: ELISA; phosphate-buffered saline: PBS; haematoxylin–eosin: H&E; Dulbecco’s modified Eagle medium: DMEM; foetal bovine serum: FBS; multiplicity of infection: MOI; Cell Counting Kit-8: CCK-8; real-time quantitative PCR: RT-qPCR; 4′;6-diamidino-2-phenylindole: DAPI; enhanced chemiluminescence: ECL; bovine serum albumin: BSA; horseradish peroxidase: HRP.
